# Case Report: Light chain amyloidosis in a patient with low-grade B-cell lymphoma

**DOI:** 10.3389/fneph.2026.1959759

**Published:** 2026-10-06

**Authors:** Alex Matthaeus, David Thomas, Hakan R. Toka

**Affiliations:** 1 Graduate Medical Education (GME), Hospital Corporation of America (HCA) Blake Florida Hospital, Bradenton, FL, United States; 2 NEPHROCOR, Bradenton, FL, United States; 3 Manatee Kidney Diseases Consultants, Bradenton, FL, United States

**Keywords:** AL amyloidosis, light-chain amyloidosis, low-grade B-cell lymphoma, non-Hodgkin lymphoma, renal amyloidosis, IgM monoclonal lambda gammopathy

## Abstract

**Background:**

Light chain (AL) amyloidosis is typically associated with plasma cell dyscrasias but can rarely arise from low-grade B-cell non-Hodgkin lymphoma. Renal involvement may present with nephrotic-range proteinuria, acute kidney injury, edema, anasarca, and hypoalbuminemia. Recognizing lymphoma-associated AL amyloidosis is essential because diagnosis requires tissue confirmation, and treatment must target the underlying clonal B-cell disorder.

**Case:**

We report a rare case of severe renal AL amyloidosis in a patient with known low-grade B-cell non-Hodgkin lymphoma previously treated with rituximab. The patient presented with witnessed syncope, progressive peripheral edema, anasarca, and hypotension. Initial evaluation showed acute kidney injury with nephrotic-range proteinuria, including a urine protein-to-creatinine ratio greater than 10 g/g. Workup for autoimmune, infectious, and malignant causes of proteinuric kidney disease revealed positive hepatitis C antibody, mildly elevated serum lambda free light chains, a mildly decreased kappa-to-lambda ratio, and serum immunofixation positive for an IgM monoclonal lambda gammopathy. Renal biopsy demonstrated severe AL amyloidosis with predominant renal involvement. Bone marrow biopsy showed low-grade B-cell non-Hodgkin lymphoma involving 85% of the marrow, with CD20-positive immunophenotyping.

**Conclusion:**

This case highlights a rare presentation of renal AL amyloidosis secondary to low-grade B-cell non-Hodgkin lymphoma. Persistent nephrotic-range proteinuria in patients with B-cell lymphoma, particularly with IgM lambda monoclonal gammopathy, should prompt evaluation for amyloidosis with renal biopsy and hematologic assessment.

## Introduction

Light chain (AL) amyloidosis is an uncommon systemic protein misfolding disorder with an estimated incidence of 9–14 cases per million person-years in the United States. The disease primarily affects older adults, with a median diagnostic age of approximately 64 years, and demonstrates a male predominance, with men comprising nearly 60% of affected individuals. AL amyloidosis arises from a clonal plasma cell or B-cell population that overproduces misfolded immunoglobulin light chains, most commonly in the setting of plasma cell dyscrasias such as multiple myeloma or monoclonal gammopathy of undetermined significance (MGUS) (1–3). Light chain amyloidosis associated with non-Hodgkin lymphoma constitutes a distinctly less prevalent pathogenic subtype, reflecting alternative lymphoid cell origins of the monoclonal light chain (8). The clinical phenotype is dictated by tissue tropism and the burden of amyloid deposition, with cardiomyopathic involvement leading to restrictive physiology, renal deposition producing nephrotic-range proteinuria and progressive renal insufficiency, and systemic manifestations such as fatigue, hypotension, or unintentional weight loss arising from multisystem infiltration (4, 5).

## Case description

An 80-year-old woman with a history of non-Hodgkin lymphoma treated with a rituximab-based regimen, heart failure with preserved ejection fraction, hypertension, and coronary artery disease presented after a witnessed syncopal episode. She described a prodrome of progressive lightheadedness and tunnel vision, followed by a brief loss of consciousness with rapid spontaneous recovery. On admission, she was hypotensive with a blood pressure of 86/58. Neurologic and cardiac evaluations, including assessment for arrhythmia and structural disease, were unrevealing. Initial laboratory testing revealed significant proteinuria. Physical examination showed significant bilateral lower-extremity pitting edema and generalized fatigue. She was admitted for further evaluation of syncope and unexplained proteinuria. Additional studies demonstrated a markedly elevated urine protein-to-creatinine ratio (UPCR), an increased serum lambda free light chain, and an IgM monoclonal gammopathy on serum immunofixation (see **Table 1**). She was diagnosed with nephrotic syndrome of uncertain etiology, and an outpatient renal biopsy was scheduled. She was also diagnosed with adrenal insufficiency and treated with hydrocortisone and fludrocortisone.

**Table 1:** Laboratory and diagnostic findings.

Parameter	Admission	Follow-up (4 weeks later)
Urinalysis (UA)	3+ proteinuria	3+ proteinuria
Creatinine (Cr)	1.8 mg/dL	1.15 mg/dL
Blood urea nitrogen (BUN)	45.0 mg/dL	21.0 mg/dL
Albumin	3.0 g/dL	3.0 g/dL
UPCR	10 g/g	7.9 g/g
C3 complement	78 mg/dL	—
C4 complement	9.7 mg/dL	—
Hepatitis C antibody	Positive	—
HCV RNA	Not detected	—
Cryoglobulins	Not detected	—
antineutrophil cytoplasmic antibodies (ANCA) (P-ANCA/C-ANCA)	Negative	—
Serum lambda free light chain	131 mg/L	—
Serum kappa free light chain	14.41 mg/L	—
Serum kappa/lambda ratio	0.11	—
Serum immunofixation	IgM monoclonal lambda gammopathy	—
Urinary free kappa light chain	—	55.97 mg/L
Urinary free lambda light chain	—	100.18 mg/L
Urine kappa/lambda ratio	—	0.56
Echocardiogram (TTE)	Ejection fraction (EF) 60–65%; normal Left ventricle (LV) thickness; trivial regurgitation	—

UPCR, urine protein-to-creatinine ratio.

## Diagnostic assessment

Renal biopsy evaluation by light microscopy, direct immunofluorescence, and electron microscopy demonstrated AL amyloidosis with lambda light-chain composition, involving the glomeruli, interstitium, arteries, and arterioles (**Figure 1**). Findings included 4% small partial cellular crescents, moderate acute tubular epithelial cell injury, moderate interstitial fibrosis, 52% global glomerulosclerosis, and moderate arteriosclerosis.

**Figure 1:**
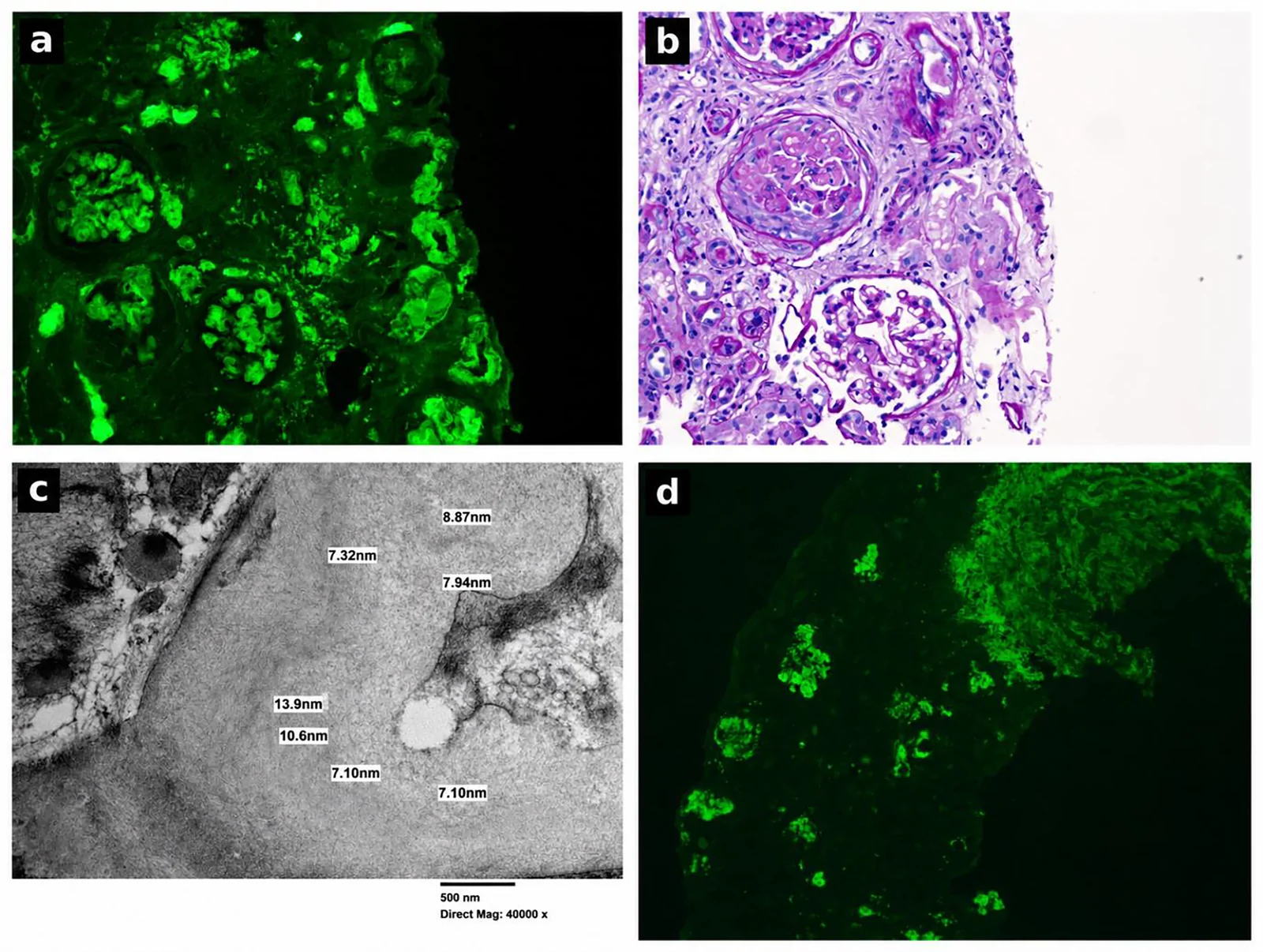
Renal biopsy findings in light chain (AL) amyloidosis. **(a)** Lambda immunofluorescence staining of renal biopsy demonstrating strong lambda light-chain restriction with bright glomerular and tubulointerstitial staining, supporting lambda-predominant light-chain deposition in the setting of AL amyloidosis. **(b)** Renal biopsy light microscopy showing amorphous eosinophilic glomerular and tubulointerstitial deposits with mesangial expansion, capillary wall involvement, and focal crescent formation (see arrow), consistent with renal amyloid deposition. **(c)** Electron microscopy of renal biopsy showing extracellular deposition of randomly oriented, non-branching fibrils measuring approximately 7–13 nm in diameter, a characteristic ultrastructural finding of amyloid deposition. **(d)** Thioflavin T fluorescence staining of renal biopsy showing strong staining of amorphous deposits involving glomeruli, tubular basement membranes, and interstitium, consistent with renal amyloid deposition.

A subsequent bone marrow biopsy revealed low-grade B-cell non-Hodgkin lymphoma involving 85% of the bone marrow. Immunophenotyping demonstrated a lambda-restricted clonal B-cell population that was positive for CD20 and negative for CD5, CD10, and CD11c. Although the immunophenotypic findings raised consideration for lymphoplasmacytic lymphoma/Waldenström macroglobulinemia, MYD88 L265P mutation testing was not performed, precluding definitive subclassification. PET imaging demonstrated lymphomatous involvement without additional abnormal uptake or lymphadenopathy.

The patient was initiated on rituximab and bendamustine for a planned six cycles. She is currently tolerating therapy well, with moderate improvement in renal function, as evidenced by a decrease in last serum creatinine to 1.15 mg/dL.

Prognosis in AL amyloidosis is primarily determined by the extent of organ involvement and disease stage. Cardiac involvement is the most significant predictor of poor outcomes, with heart failure accounting for approximately 50% of amyloidosis-related deaths. Renal failure and infection each contribute to approximately 15% of mortality. In untreated patients, progression to dialysis-dependent renal failure occurs in an estimated 60%–75% within 3 years. Earlier studies reported median survival as low as 6 months; however, advances in immunomodulatory therapies and earlier diagnosis have substantially reduced mortality and improved overall survival.

## Discussion

Several studies have highlighted a significant association between AL amyloidosis and aggressive non-Hodgkin lymphoma (NHL) subtypes, particularly diffuse large B-cell lymphoma and mantle cell lymphoma. Despite this overlap, NHL-associated AL amyloidosis remains a rare and likely underdiagnosed disease process. This is in part due to the diagnostic challenges posed by the often-overlapping symptoms of the two conditions, including fatigue, edema, and renal dysfunction. Because these clinical manifestations are non-specific, histopathological confirmation with free light chain assays followed by tissue biopsy remains essential for establishing an accurate diagnosis (5, 6). Early detection of AL amyloidosis is critical, as it helps guide management in patients who frequently have significant comorbidities that negatively impact their overall prognosis (4–6).

Importantly, AL amyloidosis represents only one entity within the broader differential diagnosis of nephrotic syndrome in patients with NHL (see **Table 2**), with only 1%–4% of amyloidosis cases being attributed to NHL. A variety of paraneoplastic and immune-mediated glomerular diseases can accompany lymphoma, including minimal change disease—one of the most common glomerulopathies seen in T-cell lymphomas—membranous nephropathy from immune complex deposition, and less frequently focal segmental glomerulosclerosis driven by podocyte injury. Other monoclonal gammopathy-related conditions, such as light-chain deposition disease or, in rare cases, cast nephropathy, may also produce heavy proteinuria. Additionally, cryoglobulinemic glomerulonephritis and immune complex membranoproliferative glomerulonephritis can occur in lymphoplasmacytic and other B-cell NHL subtypes. Distinguishing among these entities requires careful integration of clinical features, serologic testing, complement levels, and definitive renal biopsy findings. Recognizing the breadth of this differential is essential because treatment differs significantly depending on the underlying pathology (5, 6).

**Table 2:** Differential diagnosis of nephrotic syndrome in non-Hodgkin lymphoma.

Renal pathology	Associated NHL types	Mechanism/pathophysiology	Key features	Biopsy findings
Minimal change disease (MCD)	T-cell NHL, peripheral T-cell lymphoma, rare in B-cell NHL	Cytokine-mediated podocyte injury (IL-13, lymphokines)	Acute nephrotic syndrome, selective proteinuria, normal complement	Diffuse podocyte foot process effacement; light microscopy often normal
Membranous nephropathy (MN)	Various B-cell NHL subtypes	Tumor-related antigens → immune complex deposition	Nephrotic syndrome; may have microscopic hematuria	Subepithelial deposits; thickened Glomerular basement membrane (GBM); granular IgG/C3; PLA2R negative
Focal segmental glomerulosclerosis (FSGS)	Both B-cell and T-cell NHL (rare)	Podocyte injury via circulating factors; hyperfiltration injury	Nephrotic or mixed proteinuria	Segmental sclerosis of glomeruli; podocyte hypertrophy
AL amyloidosis	Lymphoplasmacytic lymphoma, Waldenström macroglobulinemia	Monoclonal light-chain misfolding → amyloid fibrils	Massive proteinuria, edema, organ enlargement	Congo red-positive deposits; apple-green birefringence
Light-chain deposition disease (LCDD)	Plasmacytoid NHL, lymphoplasmacytic lymphoma	Non-fibrillar monoclonal κ/λ chain deposition	Proteinuria with renal insufficiency	Nodular glomerulosclerosis; linear light-chain staining
Cast nephropathy	Plasmacytoid or lymphoplasmacytic variants of NHL	Overproduction of free light chains → tubular obstruction	AKI + proteinuria	Fractured casts with tubular injury
Cryoglobulinemic GN	Lymphoplasmacytic NHL, marginal zone lymphoma	Circulating cryoglobulins → immune complex GN	Proteinuria + hematuria; low C4	MPGN pattern; intracapillary ‘pseudothrombi’
Immune complex MPGN	B-cell NHL	Chronic antigen drive → immune complexes	Nephrotic or mixed syndrome; low complement	MPGN pattern with immune complex deposition
Lymphomatous renal infiltration	Aggressive B-cell NHL (DLBCL, Burkitt)	Direct infiltration of kidney parenchyma	Renomegaly, AKI; usually mild proteinuria	Sheets of malignant lymphoid cells in interstitium

NHL, non-Hodgkin lymphoma; AKI, acute kidney injury; GN, glomerulonephritis; MPGN, membranoproliferative glomerulonephritis; DLBCL, diffuse large B-cell lymphoma.

Therapeutically, while stem cell transplantation remains an option for select patients with limited comorbidities and Dara–CyBorD is widely used for AL amyloidosis, standard NHL chemotherapy regimens—such as those incorporating rituximab—may also reduce amyloid burden and improve renal outcomes when both processes coexist (4). Emerging clinical trials are evaluating novel agents and combination therapies that target both the lymphoid malignancy and the associated renal pathology, offering promising preliminary results and the potential for more personalized therapeutic strategies (6–8).

This case has several notable strengths. A multidisciplinary diagnostic approach incorporating serologic evaluation, renal biopsy with Congo red staining, serum free light chain analysis, bone marrow biopsy, and immunophenotyping enabled the timely identification of lymphoma-associated AL amyloidosis and the initiation of clone-directed therapy. In contrast to many published reports describing advanced multisystem disease or delayed diagnosis after irreversible organ damage has occurred, this patient underwent prompt evaluation for nephrotic syndrome, allowing early treatment with rituximab and bendamustine and subsequent improvement in renal function and proteinuria (5–8). However, several limitations should be acknowledged. Definitive amyloid typing by laser microdissection with mass spectrometry was not performed, although the clinicopathologic findings strongly supported AL amyloidosis. In addition, MYD88 L265P mutation testing was not obtained, precluding definitive exclusion of lymphoplasmacytic lymphoma/Waldenström macroglobulinemia, and the relatively short duration of follow-up limits assessment of long-term hematologic remission, renal recovery, and survival. Despite these limitations, this case adds to the limited body of literature describing renal AL amyloidosis associated with low-grade B-cell non-Hodgkin lymphoma and reinforces the importance of maintaining a high index of suspicion for amyloidosis in patients with lymphoma who present with nephrotic syndrome or otherwise unexplained renal dysfunction (4–6).

## Patient perspective

Although the patient had a long-standing history of non-Hodgkin lymphoma, the diagnosis of renal light chain amyloidosis as a manifestation of disease progression was unexpected. She expressed appreciation for the prompt medical evaluation of her condition and the clear, open communication provided by the multidisciplinary team involved in her care. She tolerated treatment with rituximab and bendamustine well, experiencing only minimal side effects, and has noted a significant improvement in her peripheral edema since initiating therapy. She remains optimistic about her prognosis and is hopeful that ongoing treatment will continue to minimize her light chain amyloidosis disease burden while allowing her to maintain a good quality of life.

## Data Availability

The original contributions presented in the study are included in the article/Supplementary Material. Further inquiries can be directed to the corresponding author/s.
